# Clinical outcomes of Pfizer‐BioNTech COVID‐19 vaccine in children and adolescents: A systematic review

**DOI:** 10.1002/hsr2.740

**Published:** 2022-07-20

**Authors:** Ahmad R. Al‐Qudimat, Raed M. Al‐Zoubi, Mai Elaarag, Abdulqadir J. Nashwan, Afaf K. Hamze, Hiba Bawadi, Aksam Yassin, Aseel Assim, Omar M. Aboumarzouk, Ahmad Zarour, Abdulla A. Al‐Ansari

**Affiliations:** ^1^ Hamad Medical Corporation Doha Qatar; ^2^ Department of Biomedical Sciences, College of Health Sciences, QU‐Health Qatar University Doha Qatar; ^3^ Department of Chemistry Jordan University of Science and Technology Irbid Jordan; ^4^ Center of Medicine and Health Sciences Dresden International University Dresden Germany; ^5^ College of Medicine, QU‐Health Qatar University Doha Qatar; ^6^ School of Medicine, Dentistry and Nursing The University of Glasgow Glasgow UK

**Keywords:** adolescents, BNT162b2, children, COVID‐19, Pfizer‐BioNTech, symptoms, vaccine

## Abstract

**Background & Aims:**

The BioNTech‐Pfizer vaccine is the only vaccine offered to children among all available vaccines. However, limited evidence is available about the clinical outcomes of COVID‐19 vaccines, especially among children and adolescents. This review offers a comprehensive and up‐to‐date overview of the BioNTech‐Pfizer vaccine's current information on children and adolescents.

**Methods:**

The review was conducted following the PRISMA guidelines; a comprehensive search was performed in PubMed, Scopus, MEDLINE, and EMBASE databases for research publications COVID‐19 published between December 2019 and October 2021. All studies reporting on the outcomes of vaccinating children in their respective institutes were included.

**Results:**

A total of 78 vaccinated children and adolescents from six studies were included. The majority of symptomatic vaccinated pediatrics were males (71%). The mean age was 15.6 years, and the BMI was 24.1. The most common clinical symptoms were found in chest pain (35%), fever (32%), and myalgia (17%). The most common cardiac symptom in the EKG results was ST elevation, and 35% of vaccinated pediatrics had elevated serum troponin. The hospitalization, including ICU admission, was lower than in unvaccinated groups. Statistically significant associations (*p* ≤ 0.05) were found in two symptoms (fever and headache) between the vaccinated and nonvaccinated pediatric groups.

**Conclusions:**

Although we found better outcomes in the vaccinated group versus the nonvaccinated pediatric group, more studies are still crucial to further understand the specific etiology underlying postvaccination, particularly myocarditis, psychological impact, and other cardiac clinical symptoms in children and adolescents after receiving the BioNTech‐Pfizer vaccine.

## 1 INTRODUCTION

The severe acute respiratory syndrome coronavirus 2 (SARS‐CoV‐2) has swiftly spread around the world, causing a global pandemic (COVID‐19) declared by the World Health Organization (WHO). COVID‐19 resulted in a significant increase in morbidity and death, as well as significant economic damage.^1^ As of November 20, 2021, there have been more than 257 million confirmed cases and more than five million death cases reported to WHO.

The highly contagious coronavirus strain has overwhelmed the global healthcare systems for the third time in this century. The first coronavirus pandemic was started in 2002 by a severe acute respiratory syndrome coronavirus (SARS‐CoV‐1). As a result, healthcare workers were at a higher risk of developing the disease than others in the population.^2^ In 2012, the Middle East respiratory syndrome coronavirus (MERS‐CoV) spread globally, causing the second outbreak. According to the WHO, the MERS‐CoV virus is still circulating with a 35% fatality rate compared to 9.5% for SARS‐CoV‐1.^1^ SARS‐CoV‐1 has a case fatality rate of 2%–3%, according to reports.^3^

Nowadays, the world suffers from the third coronavirus infection (SARS‐CoV‐2) and causes severe acute respiratory syndrome.^4^, ^5^, ^6^ It began back in December 2019, when Chinese health officials in Wuhan discovered severe respiratory distress due to pneumonia in a cluster of people. On January 7, 2020, the new coronavirus strain dubbed novel coronavirus 2019 came from these patient clusters (2019‐nCoV).

In the united states (US) alone, 9%–12% of diagnosed patients with COVID‐19 were children.^7^ About 90% of children who tested positive were asymptomatic or had mild‐to‐moderate symptoms. Only 15 children required critical care in a survey of 2572 pediatric cases, with three deaths documented.^8^ Another study across North America found that 18 of 48 children brought to ICUs required invasive ventilation, where 16 children survived and two died.^9^ Children under the age of 1 year, as well as those with additional comorbidities or underlying diseases, were found to be at higher risk of severe illness.^10^, ^11^, ^12^ It is suggested that COVID‐19 in the pediatric population was less severe compared to cases in adults, and diagnosed children had different symptoms than adults do.^13^, ^14^ Interestingly, children might not have coughing or fever as frequently reported in adults.^14^

Vaccinations and preventive measures are crucial for all ages to protect children from new variants of this virus‐like Delta and Omicron and for patients with comorbidities and to have more control over disease transmission.

Accordingly, WHO granted global emergency approval of vaccines.^15^ The fast development of COVID‐19 vaccines raised many concerns and questions. In 2020 and during this pandemic, the messenger RNA (mRNA) type of vaccines have been used on humans and showed a significant efficacy rate.^16^, ^17^, ^18^, ^19^, ^20^

The BioNTech‐Pfizer COVID‐19 vaccine is developed from a single‐stranded mRNA made in vitro transcription from a DNA template that encodes the viral spike protein.^21^

This review aims to offer healthcare workers and non‐healthcare workers a comprehensive and up‐to‐date overview of the currently available information on the severity of the BioNTech‐Pfizer vaccine in children and adolescents. Also, to provide the scientific readers with useful data that can aid in early recognition and effective prevention and management of children affected by COVID‐19 and the BioNTech‐Pfizer vaccine.

## 2 METHODS

A systematic review was conducted in accordance with the Preferred Reporting Items for Systematic Reviews (PRISMA) guidelines,^22^ and a comprehensive search was performed in PubMed, Scopus, MEDLINE, and EMBASE Databases for research publications on COVID‐19 published between December 2019 and October 2021 using the terms COVID‐19, Pediatrics, children, adolescents, SARS‐CoV‐2, Epidemiology, and Vaccine. Phrases used for the Medical Subject Heading (MeSH) search included: (((“COVID‐19” [MeSH]) and “Vaccine” [MeSH]) and “Children” [MeSH]) and “adolescents” [MeSH]) and “Pfizer” [MeSH]. We focused on original research, case reports, case series, and vaccination side‐effect by authoritative health institutions. All records were retrieved, including original articles, letters to the editor, editorials, and case reports, in English, and records with English translation were downloaded and reviewed. Given the current scarcity of evidence, preprints, in‐press papers, and accepted‐for‐publication research were also considered.

## 3 INCLUSION AND EXCLUSION CRITERIA

Studies were included for the systematic review if they met the following criteria (a) infants, children, and adolescents who aged <18 and had COVID‐19 BioNTech‐Pfizer vaccine; (b) children and adolescents who got infected with COVID‐19, either confirmed microbiologically or clinically; exclusion criteria were any patient of age ≥18 years.

## 4 SEARCH STRATEGY

### 4.1 Study selection and data collection

Through abstract screening, the previously indicated inclusion criteria were applied to choose possibly relevant articles. Two authors (A.R.A. and O.M.R.) assessed full texts of relevant articles and screened them according to the inclusion criteria. Where there was a difference of opinions, conversations with the senior authors (O.M.A. and R.M.A.) were held until a consensus was established.

### 4.2 Data extraction

We extracted as many relevant variables as possible from the information provided (age, date, etc.) and according to the main stratification variable, author, country, data source, age range, study timeframe, baseline population group, outcome (symptoms after vaccination, severity of symptoms), total sample, others. We additionally included another category of the length of stay in the hospital or ICU. We retrieved data on the age who are confirmed vaccinated by BioNTech‐Pfizer COVID‐19, confirmed symptoms after vaccination, and length of stay in hospitals or ICU from publicly available data. All studies included in this review were published in the United States of America (USA).

## 5 RESULTS

**Figure 1:**
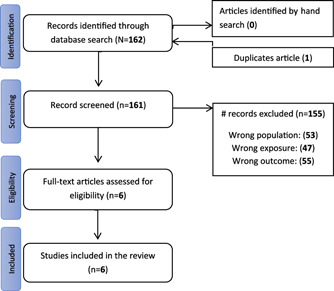
PRISMA diagram of literature search

The initial literature search retrieved 162 potentially relevant studies. After initial screening, one study was excluded due to duplication, and 155 studies were further excluded after reviewing the title, abstract, or not meeting the eligibility criteria. Only six studies were relevant and included in the review (Figure 1).

### 5.1 Risk of bias assessment

Since only nonrandomized studies are included in this review, one risk of bias assessment was performed. Two researchers worked together to complete the risk of bias assessment; after that, a third researcher compared and integrated the findings of this assessment. If there was any disagreement, the fourth investigator acted as a tie‐breaker. The Risk of Bias in Non‐Randomized Studies of Interventions (ROBINS‐I) tool was used to perform the risk assessment.

According to the traffic light plot of the risk of bias assessment (nonrandomized studies), 81% had a low risk of bias (Table 1). Two out of six studies were found to have a moderate risk of bias. None of the studies were found to have a critical risk of bias.

**Table 1:** Traffic light plot of risk of bias assessment (nonrandomized studies)

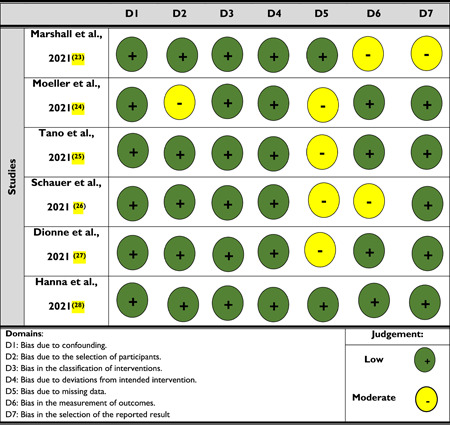

### 5.2 Ethical considerations

The ethical review was deemed unnecessary due to the nature of this study (a systematic review). However, all data were confidentiality observed and protected. All data used in this review were accessed and evaluated only by the researchers and kept on personal password‐protected computers. The protocol for this systematic review was registered in the International Prospective Register of Systematic Reviews (PROSPERO) with a unique ID: CRD42021297129.

### 5.3 Study characteristics and cumulative analysis

In total, six reports were included in this study.^23^, ^24^, ^25^, ^26^, ^27^, ^28^ There were no randomized trials, and all studies were observational (retrospective, case series, and case report) as shown in Table 2. A total of 78 vaccinated children and adolescents were included. Gender was provided for all patients and included 55 males and 23 females. The mean age was 15.6, ranging between 14.5 and 18 years old. A total of 66 out of 78 patients were found to be White and the dominant race in the analysis. One median was provided by one study and was not calculated in the analysis. BMI was included in only two studies.

**Table 2:** Summary of relevant research studies and characteristics of vaccinated children and adolescents after BioNTech‐Pfizer COVID‐19 vaccine

Author, Year	Country	Study Design	Pediatric patients	Enrollment period	Age (mean)	Gender (M/F)	Race	BMI (mean)
Marshall et al. (2021)^23^	USA	Case report	7	(April to May 2021)	16.7	7/0	6 White, 1 Hispanic	22
Moeller et al. (2021)^24^	USA	Retrospective	33	(May to June 2021)	14.5	12/21	30 White, 1 black, 2 others	N_S_
Tano et al. (2021)^25^	USA	Case series	8	(May 2021)	16.6	8/0	8 white	26.2
Schauer et al. (2021)^26^	USA	Retrospective	13	(April to June 2021)	15.1	12/1	10 White, 2 Asian, 1 Alaska native	N_S_
Dionne et al. (2021)^27^	USA	Case series	15	(May to July 2021)	15 (12–18)^a^	14/1	10 White, 5 others	N_S_
Hanna et al. (2021)^28^	USA	Case report	2	(May 2021)	15	2/0	2 White	N_S_
Total			78		14.5–18^b^	55/23	66 White, 1 black, 1 Hispanic, others	

Abbreviations: BMI, body mass index; N_S_, not stated.

^a^ Median.

^b^ Range.

The clinical symptoms of the BioNTech‐Pfizer vaccine on vaccinated children and adolescents are demonstrated in Table 3. The symptoms of vaccinated children and adolescents were listed and summarized. The most common clinical symptoms were found in chest pain (35%), fever (32%), myalgia/muscle pain (17%), headache (8%), fatigue (13%), SOB (17%), vomiting (5%), nausea (4%), malaise (4%) and local pain (3%). The number of days to till show symptoms after administering the COVID‐19 vaccine ranged from 1 to 6 days.

**Table 3:** Clinical signs and symptoms of children and adolescents after BioNTech‐Pfizer COVID‐19 vaccine

Author, Year	Children and adolescents' clinical signs and symptoms after second dose of BioNTech‐Pfizer COVID‐19 vaccine (BNT162b2)																		
	Patients No.	Gender (M/F)	Days to symptoms	Fever	Fatigue	Headache	Nausea	Vomiting	SOB	Myalgia	Weakness/Malaise	Chest pain	Local pain	Abnormal EKG/ECG	Abnormal Echocardiogram	Cardiac MRI			Abnormal troponin
																MRI No.	Cardiac MRI edema	Cardiac MRI LGE	
Marshall et al. (2021)^23^	7	7/0	1–4	3	3	1	3	2	2	1	1	7	2	7	2	7	6	6	3
Moeller et al. (2021)^24^	33	12/21	N_A_	N_A_	N_A_	N_A_	N_A_	N_A_	N_A_	N_A_	N_A_	N_A_	N_A_	N_A_	N_A_	N_A_	N_A_	N_A_	N_A_
Tano et al. (2021)^25^	8	8/0	1–4	7	1	2	N_S_	1	6	N_S_	N_S_	5	N_S_	6	0	3	3	3	8
Schauer et al. (2021)^26^	13	12/1	2–4	5	N_S_	3	N_S_	1	5	4	2	N_S_	N_S_	8	2	13	13	13	13
Dionne et al. (2021)^27^	15	14/1	1–6	10	6	N_S_	N_S_	N_S_	N_S_	8	N_S_	15	N_S_	9	1	15	0	12	3
Hanna et al. (2021)^28^	2	2/0	≤1	N_S_	N_S_	N_S_	N_S_	1	N_S_	N_S_	N_S_	N_S_	N_S_	N_S_	N_S_	N_S_	N_S_	N_S_	N_S_
Total	78	55/23	1–6	25	10	6	3	4	13	13	3	27	2	30	5	38	22	34	27

Abbreviations: BMI, body mass index; MRI, magnetic resonance imaging; N_A_, not applicable; N_S_, not stated.

For instance, Hanna et al. reported a study of two pediatric patients with IgAN presenting with microscopic hematuria less than 24 h after receiving the BioNTech‐Pfizer vaccine. The authors found a 13‐year‐old male patient had a history of IgAN and type 1 diabetes, whereas the other male patient in this study was found healthy with no medical history before receiving the vaccine.^28^ Children and adolescents' clinical cardiac symptoms are also demonstrated in Table 3. It included the abnormal EKG and echocardiogram, abnormal cardiac MRI findings including pericardial effusion, and high troponin levels. It is worth noting that troponin levels were elevated in 35% of patients.^23^, ^25^, ^26^, ^27^ However, no testing showed pericardial effusion. The most common EKG result was ST elevation. Marshall et al. reported atrioventricular dissociation with junctional escape rhythm, T‐wave abnormality, and sinus bradycardia.^23^ Moreover, Tano et al. found in their study that among patients who had EKG, two had RP depression, one had abnormal waves, and one had ST depression and conduction delay.^25^ The abnormal echocardiogram outcomes were included in three studies.^23^, ^26^, ^27^ For instance, Marshall et al. reported in their study that only one patient had mildly depressed RV and LV systolic function (LVEF 47%) and basal lateral and posterior strain.^23^ Ejection fraction was reported by Schauer et al.^26^ and Tano et al.^25^ for 13 and 8 patients, respectively. Moeller et al. reported the psychological symptoms of children and adolescents after having the BioNTech‐Pfizer vaccine.^24^ Their study included 33 pediatric patients, three of them had anxiety, three had attention deficit or hyperactivity, four had disruptive mood dysregulation disorder, and 18 of them had a major depressive disorder or suicidal ideation.

The hospitalization of 78 vaccinated children and adolescents was also investigated. Among the vaccinated pediatrics during the period from April to July 2021, and according to six studies,^23^, ^24^, ^25^, ^26^, ^27^, ^28^ for whom clinical data are completed and available, 45 (58%) were hospitalized, and five (6.4%) were admitted to ICU. The range of hospital stay for vaccinated pediatrics was found to be 1–6 days.

The difference in clinical symptoms/signs between vaccinated and nonvaccinated pediatrics is shown in Table 4. Symptoms were found milder in vaccinated pediatrics compared to the diagnosed COVID‐19 pediatrics. For instance, none of the vaccinated pediatrics had cough symptoms compared to 51% (150/297) of nonvaccinated pediatrics. Only 32% (25/78) of vaccinated pediatrics had fever compared to 55% (163/297) of the nonvaccinated group. Seventeen percent (13/78) had SOB compared to 13% (39/297). Seventeen percent (13/78) of vaccinated pediatrics had muscle pain compared to 22% (66/297) for nonvaccinated pediatrics. Headache was only reported in 8% (6/78) compared to 27% (81/297) for the nonvaccinated group. Lastly, 9% (7/78) of the vaccinated group had nausea/vomiting compared to 10% (31/297).

**Table 4:** Percentage of signs and symptoms among 78 vaccinated pediatric (age < 18 years) with BioNTech‐Pfizer vaccine and 297 nonvaccinated pediatric (age < 18 years, lab‐confirmed COVID‐19) in North America.

Sign/symptom	No. (%) of pediatric patients		
	Vaccinated (*n* = 78) [23–28]	Nonvaccinated (*n* = 297) [5]	*p* value
Fever	25 (32%)	163 (55%)	<0.001
Cough	0 (0%)	150 (51%)	‐
SOB	13 (17%)	39 (13%)	0.421
Muscle pain	13 (17%)	66 (22%)	0.284
Headache	6 (8%)	81 (27%)	<0.001
Nausea/vomiting	7 (9%)	31 (10**%**)	0.703

A Pearson's *χ* ^2^ test showed statistically significant associations (*p* ≤ 0.05) in two symptoms (fever and headache) between the vaccinated and nonvaccinated groups (Table 4). Additionally, no cough symptom was reported for the vaccinated group compared to 51% (150/297). There was a decrease in fever in the vaccinated group (from a mean of 163 [55%] to 25 [32%], *p* ≤ 0.001) and in headache from (a mean of 81 [27%] to 6 [8%], *p* ≤ 0.001). No other statistically significant associations are observed with other symptoms between the two groups. For instance, a mean of 13 (17%) for both SOB (*p* = 0.421) and muscle pain (*p* = 0.284), 7 (9%) for nausea/vomiting (*p* = 0.703) for the vaccinated group compared to a mean 39 (13%), 66 (22%) and 31 (10%) in the nonvaccinated group, respectively.

## DISCUSSION

This article is a comprehensive review of up‐to‐date clinical findings of the BioNTech‐Pfizer vaccine on children and adolescents. We aimed to provide knowledge through the evaluation of existing evidence regarding the BioNTech‐Pfizer vaccine on children and adolescents. All published articles were found using a thorough search strategy on three databases (PubMed, Scopus, MEDLINE, and EMBASE Databases) on October 17, 2021, and without restrictions to language or region. Although children and adolescents have higher levels of antibodies and their immune system response to pathogens is different than adults and most of them have less severe or asymptomatic COVID‐19 cases,^13^ some symptoms were reported for this group after vaccination. The preliminary clinical outcomes of vaccinated children and adolescents suggest common mild symptoms such as fever and headache but also worth noting that some other symptoms such as chest pain is reported, which require further studies and health concerns. The final results of signs and symptoms included 78 vaccinated children and adolescents from six studies.

A recent report from Morbidity and Mortality Weekly Report (MMWR) revealed that the hospitalization of COVID‐19‐related pediatrics during the week ending August 14, 2021 was approximately five times the rate of the week ending June 26, 2021.^29^ Moreover, the authors showed that among the children of age 0–4 years during the week ending of August 14, 2021 was approximately 10 times higher than of the same age by the week ending of June 26, 2021. In addition, the hospitalization of unvaccinated adolescents of age 12–17 years during the period from June 20 to July 31, 2021 was 10.1 times higher than the vaccinated adolescents. The rate of hospitalization for COVID‐19–associated pediatrics increased rapidly with the predominance of the Delta variant from June 26 to August 14, 2021. In this study, we found that the hospitalization of vaccinated pediatrics (78 patients) in six studies and from April to July 2021 was less compared to the unvaccinated group. A total of 37 (47%) were hospitalized with a mean range of hospital stay between 1 and 6 days for the vaccinated pediatrics. ICU admission was reported only for five (6.4%) and in only one article with ICU stay for 2–5 days.

It is also important to look at how the patients were doing after being discharged from the hospital. In the study by Schauer et al., four patients complained of intermittent chest pain at follow‐up, however, no identifiable abnormalities were found upon evaluation and no therapy or intervention was required.^26^ The other cardiac study by Dionne et al., had a follow‐up period at 1–3 days after discharge, where 11 patients had resolution of symptoms, one patient had persistent borderline low LV systolic function on echocardiogram, three patients had mildly elevated troponin levels, and one patient showed nonsustained ventricular tachycardia on the ambulatory monitor.^27^ Lastly, the study by Marshall et al., recorded follow‐ups for all seven patients included in their study; for Patient 1, during his treatment, he was followed up 3 weeks later, where troponin returned to normal, Patients 2, 3, and 4 were followed up after 1 week and had the normal presentation of all symptoms, Patient 5 was followed up 4 days later and revealed normal echocardiogram, however, ECG revealed diffuse T‐wave abnormalities, Patient 6 had no follow‐up, and finally, Patient 7 was followed up 13 days later and reported chest pain with exertion however, appeared well and had a normal presentation of the rest of the symptoms.^23^ The study by Hanna et al., did not specify their follow‐ups for each patient and the studies by Tano et al., and Moeller et al., did not have follow‐ups recorded.

Pfizer performed a phase III clinical trial to study the effectiveness of their vaccine. In this study, 2260 children aged from 12 to 15 years were enrolled, only 18 cases of COVID‐19 were recorded for the placebo group (*n* = 1129) and none were reported for the vaccinated group (*n* = 1131). Accordingly, the study led to FDA approval of expanding the use to children aged 12–15 years.^21^ Pfizer announced that their COVID‐19 vaccine will be 100% efficient in children aged 12–15.^30^ This emergency use authorization by the FDA to include children 12–15 years of age was a significant step in our fight against this virus. Currently, trial studies are still ongoing to further test the vaccine's efficacy on younger children aged 6 months to 11 years.^30^

### 1 Clinical symptoms

The most common clinical symptoms of vaccinated pediatric patients were found that 27 had chest pain (35%), 25 had a fever (32%), 6 had a headache (8%), and 10 had fatigue (13%). All other symptoms are summarized in Table 3. In addition, the majority (71%) of symptomatic vaccinated pediatrics were males. The average length of hospitalization is 1–6 days. Although a number of diagnosed pediatrics with COVID‐19 required ICU level care, the number of pediatrics who need ICU level care after vaccinations was only reported in one study to date.^23^

Galindo et al. reported a study on the most common COVID‐19 symptoms among pediatrics.^31^ The authors looked up at 333 pediatric patients who were diagnosed with COVID‐19 and found that fever, cough, and sore throat were listed as the most common symptoms. The signs and symptoms of the COVID‐19 pediatrics were found to be noticeable to those who had the BioNTech‐Pfizer vaccine. The difference between vaccinated and nonvaccinated pediatrics is summarized in Table 4. For instance, fever presented in only 32% of vaccinated pediatrics compared to 56% of nonvaccinated and diagnosed COVID‐19 pediatrics. No cough symptom was reported for the vaccinated group compared to 55% for nonvaccinated pediatrics. Seventeen percent of vaccinated pediatrics had muscle pain compared to 22% of nonvaccinated pediatrics. All other signs were also found milder in the vaccinated group. Statistically significant associations (*p* ≤ 0.05) were found in two symptoms (fever and headache) between the vaccinated and nonvaccinated pediatric groups (Table 4). Fever and headache symptoms were found highly significant between the two groups (*p* ≤ 0.001).

A substantial percentage of children who were diagnosed with COVID‐19 are asymptomatic. However, fever, nasal congestion/rhinorrhea, dyspnea, loss of smell and taste, cough, sore throat, diarrhea, nausea/vomiting, weariness, headache, myalgia, and poor feeding/poor appetite are the most common symptoms.^13^, ^14^ Ji et al. reported the only presenting diarrhea symptom in only one pediatric case.^32^ Additionally, Wei et al. reported that only 50% of nine hospitalized newborns in China with confirmed COVID‐19 had fever.^33^

It is worth noting that vaccinated pediatrics presented clinical cardiac symptoms after the BioNTech‐Pfizer vaccine, which were not reported before as one of the symptoms for nonvaccinated diagnosed COVID‐19 pediatric and required more studies and health concerns.

### 2 Cardiac outcomes

COVID‐19 patients are commonly diagnosed with acute cardiac injury.^34^ Li et al. reported that 15%–44% of COVID‐19 patients had an incidence of cardiac injury, which was significantly larger than the prevalence found in congenital valvular disease (CVD) (5%–15%).^35^ This complied with a study published by Guo et al.,^36^ which revealed that the risk of mortality from cardiac injury was substantially higher than the risk of death from pre‐existing CVD. Although it was common in adults and elderly COVID‐19 patients, no cases were reported for COVID‐19 pediatrics. Interestingly, our findings on pediatrics who had the BioNTech‐Pfizer vaccine showed that ST‐segment elevation is the most common cardiac symptom in the ECG results.^14^, ^16^, ^17^, ^18^ The authors analyzed all vaccinated pediatric cardiac symptoms and revealed no significant risk of mortality among patients (Table 3).

Troponin was also reported by Shi et al. as an independent risk factor and a key biomarker for a sign of death among COVID‐19 patients.^37^ The elevated troponin levels for vaccinated pediatrics were also measured. It indicated an increased risk of cardiac injury in children who had the BioNTech‐Pfizer vaccine, but with no evidence of increased risk of mortality.

### 3 Psychological outcomes

The prevalence of depression and anxiety in diagnosed COVID‐19 patients are documented and ranged from 22.6%−43.7% to 18.9%−37.4%, respectively.^38^, ^39^, ^40^ For instance, Xie et al. reported a survey on home confinement among children during the COVID‐19 pandemic. The authors found that among 2330 students (1012 males), 403 students (22.6%) reported depression and anxiety symptoms.^38^ Across sectional study of 8079 participants was reported by Chen and coworkers among Chinese students (12–18 years) during the COVID‐19 pandemic to assess depressive and anxiety symptoms. It revealed a prevalence of 43.7%, 37.4%, and 31.3% of depressive symptoms, anxiety symptoms, and a combination of depressive and anxiety symptoms, respectively.^39^ Additionally, Liu et al. published a report on psychological and behavioral changes among a cluster of pneumonia patients, especially young people during the COVID‐19 pandemic. They found that patients had more state anxiety than trait anxiety (15.8% vs. 4.0%), 10.1% suffered from phobia, and depression was found among 27.1% of respondents with 7.7% having psychological abnormalities.^40^ The prevalence of depression and anxiety was also found in vaccinated children and adolescents with the BioNTech‐Pfizer vaccine. It showed the prevalence of 54.5% and 11% of depression and anxiety among vaccinated children and adolescents, respectively.

### 4 Strengths and limitations

This review focused on vaccinated children and adolescents and provided a comprehensive review and synthesis of the evidence on the BioNTech‐Pfizer vaccine's symptoms among children and adolescents. There are several limitations to this systematic review. To begin with, the included studies had low to intermediate methodological quality in terms of their sampling method, measurement validity, and statistical analysis. These points must be considered while evaluating the results. Second, we omitted a few studies from our analyses due to the lack of and/or ambiguous information. These studies reported outcomes of the vaccine, however, did not differentiate between adult and pediatric populations and the results could not be extrapolated. Nonetheless, despite these limitations, this is the first review looking at the pediatric population exclusively. Furthermore, the search and inclusion are a representation of the current published literature and should be considered the most recent and up‐to‐date evidence collectedly found.

## CONCLUSION

Humankind and the entire world are still suffering from the third coronavirus infection (SARS‐CoV‐2), causing severe acute respiratory syndrome and bringing countless morbidity, mortality, and a great economic disaster. With the new Delta andOmicron variants of this coronavirus, more preventive measures and procedures, including more vaccinations for children and adolescents, are crucial and highly recommended to reduce transmission, hospitalization, and adverse clinical outcomes. Although we found good outcomes and statistically significant associations in symptoms between vaccinated and nonvaccinated pediatrics, more studies are still crucial to understand the specific etiology further underlying postvaccination, particularly myocarditis, psychological impact, and other cardiac clinical symptoms on children and adolescents after receiving the BioNTech‐Pfizer vaccine.

## AUTHOR CONTRIBUTIONS

**Omar M. Aboumarzouk**: Conceptualization. **Ahmad R. Al‐Qudimat**, **Mai Elaarag**, and **Afaf K. Hamze**: Data curation, Methodology, Writing – original draft, Writing – review and editing. **Aksam Yassin**, **Aseel Assim**, **Omar M. Aboumarzouk**, **Ahmad R. Al‐Qudimat**, **Abdulqadir J. Nashwan**, **Raed M. Al‐Zoubi**, **Aseel Assim**, **Ahmad Zarour**, and **Hiba Bawadi**: Methodology, Writing – original draft, Writing – review and editing.

All authors have read and approved the final version of the manuscript [CORRESPONDING AUTHOR or MANUSCRIPT GUARANTOR] had full access to all of the data in this study and takes complete responsibility for the integrity of the data and the accuracy of the data analysis.

## CONFLICT OF INTEREST

The author declares no conflict of interest.

The lead author affirms that this manuscript is an honest, accurate, and transparent account of the study being reported; that no important aspects of the study have been omitted; and that any discrepancies from the study as planned (and, if relevant, registered) have been explained.

## Data Availability

All data generated during this study are included in this published article.
